# Lipide: Diagnostik und Therapie bei Diabetes mellitus (Update 2023)

**DOI:** 10.1007/s00508-023-02166-8

**Published:** 2023-04-20

**Authors:** Thomas C. Wascher, Bernhard Paulweber, Hermann Toplak, Christoph H. Saely, Heinz Drexel, Bernhard Föger, Friedrich Hoppichler, Thomas Stulnig, Harald Stingl, Martin Clodi, Ausschuss Leitlinien

**Affiliations:** 1 1 Medizinische Abteilung, Hanuschkrankenhaus, Wien, Österreich; 2 Universitätsklinik für Innere Medizin 1, Landeskrankenhaus Salzburg – Universitätsklinikum, Paracelsus Medizinische Privatuniversität, Salzburg, Österreich; 3 Universitätsklinik für Innere Medizin, Medizinische Universität Graz, Graz, Österreich; 4 Abteilung für Innere Medizin I/VIVIT Institut, Landeskrankenhaus Feldkirch, Feldkirch, Österreich; 5 Abteilung für Innere Medizin, Landeskrankenhaus Bregenz, , Lehrkrankenhaus der Medizinische Universitäten Innsbruck und Wien, Bregenz, Österreich; 6 Abteilung für Innere Medizin, A.ö. Krankenhaus der Barmherzigen Brüder, SIPCAN Institut, Salzburg, Österreich; 7 3. Medizinische Abteilung mit Stoffwechselerkrankungen und Nephrologie, Klinik Hietzing, Wien, Österreich; 8 Abteilung für Innere Medizin, Landesklinikum Melk, Melk, Österreich; 9 CMR – Institute for Cardiovascular and Metabolic Research, Johannes Kepler Universität Linz (JKU Linz), Altenberger Straße 69, 4040 Linz, Österreich

**Keywords:** Hyperlipidämie, Statine, Diabetes mellitus, Hyperlipidemia, Statins, Diabetes mellitus

## Abstract

Hyper- und Dyslipidämie tragen zur kardiovaskulären Morbidität und Mortalität diabetischer Patienten bei. Überzeugende Daten zeigen, dass eine medikamentöse Therapie mit Statinen, Ezetimibe und PCSK9 Hemmern das kardiovaskuläre Risiko von Patienten mit Diabetes senken kann. Der vorliegende Artikel stellt die Behandlungsvorschläge der Österreichischen Diabetes Gesellschaft zum Einsatz lipidsenkender Medikamente dar.

## Abstract (en)

Hyper- and dyslipidemia contribute to cardiovascular morbidity and mortality in diabetic patients. Pharmacological therapy to lower LDL cholesterol has convincingly shown to reduce cardiovascular risk in diabetic patients. The present article represents the recommendations of the Austrian Diabetes Association for the use of lipid-lowering drugs in diabetic patients according to current scientific evidence.

## Grundsatz Statement

Unabhängig vom Ausmaß der Hypercholesterinämie haben Patienten mit Diabetes gegenüber Patienten ohne Diabetes ein deutlich erhöhtes kardiovaskuläres Risiko; kardiovaskuläre Erkrankungen sind die häufigste Todesursache bei Patienten mit Diabetes.

Aufgrund der klaren Datenlage zum kardiovaskulären Risiko bei Diabetes werden Patienten mit Diabetes mellitus in den Leitlinien der ESC in die Risikokategorie „hohes“^1^ oder „sehr hohes“^2^ kardiovaskuläres Risiko eingeordnet. Dies gilt unabhängig vom Typ des Diabetes. Entsprechend der Studienlage betonen die Leitlinien des American College Clinical Endocrinologists ein „extrem hohes“ Risiko für Patienten mit Diabetes mellitus mit klinisch manifester kardiovaskulärer Erkrankung.

## Lipidstatus

Folgende Parameter sind Bestandteil einer kompletten Lipiddiagnostik und sollten unbedingt erhoben werden:

- Gesamtcholesterin
- Triglyzeride
- HDL-Cholesterin
- LDL-Cholesterin
- Nicht-HDL Cholesterin (sollte bei Triglyzeriden > 200 als Therapieziel verwendet werden)
- Lp(a) sollte einmalig bestimmt werden
- Die Bestimmung von Apo B

## Indikation zur medikamentösen Therapie

Grundsätzlich qualifizieren sich auf Basis der zu erreichenden Zielwerte die meisten Patienten mit Typ 2 Diabetes und Typ 1 Diabetes für eine lipidsenkende Therapie. Unter 40jährige Patienten mit Diabetes, die keine Diabetes-Komplikationen haben, keine weiteren Risikofaktoren, und ein LDL-C < 100 mg/dl benötigen möglicherweise keine lipidsenkende Therapie.

## Therapieziele

Unter medikamentöser Lipid-senkender Therapie sollen Lipidwerte wie in Tab. 1 dargestellt angestrebt werden.

**Table 1:** 

Sehr hohes Risiko	LDL Reduktion ≥ 50 % des Ausgangswertes *und* ein LDL Ziel < 55 mg/dl	Diabetes mit manifester Atherosklerose; Diabetes mit Endorganerkrankung (Mikroalbuminurie, Retinopathie, Neuropathie) oder zumindest mit 3 weiteren Risikofaktoren; Typ‑1 Diabetes mit früher Manifestation und > 20 Jahren Dauer
Hohes Risiko	LDL Reduktion ≥ 50 % des Ausgangswertes *und* ein LDL Ziel < 70 mg/dl	Diabetes ohne Endorganerkrankung aber einer Krankheitsdauer von > 10 Jahren oder zumindest mit einem weiteren Risikofaktor
Mittleres Risiko	LDL Ziel < 100 mg/dl	Junge Patienten (DM-1 < 35 Jahre, DM-2 < 50 Jahre) ohne weiteren Risikofaktor

Das primäre Ziel der Therapie ist das LDL-Cholesterin (Evidenzklasse A).

Ein sekundäres Therapieziel stellt bei Triglyzeriden > 200 mg/dl das Nicht-HDL-Cholesterin dar (Evidenzklasse B).

## Initiale Therapie und Kombinationstherapien

In den meisten Fällen wird ein Statin zur initialen Therapie herangezogen werden. Die initiale Auswahl ist jedenfalls aber vom Lipidstatus abhängig.

Wenn nach Einleitung einer Statintherapie der TG-Wert nicht unter 200 mg/dl sinkt, kann die zusätzliche Gabe eines Fibrates erwogen werden.

Eine Fibrattherapie sollte bei extrem hohen TG-Werten (900 mg/dl) in Erwägung gezogen werden.

Als Startdosis sollte bei Statinen mit evidenzbasierten Dosierungen (äquivalent zu zumindest 20 mg Atorvastatin oder 10 mg Rosuvastatin) begonnen werden. Eine Statintherapie sollte nach o. a. Zielwerten bzw. bis zur höchsten tolerierten Dosis gesteigert werden (Evidenzklasse A).

Möglichkeiten der sequentiellen Erweiterung einer Statintherapie sind bei nicht Erreichen des Therapiezieles und/oder Statinunverträglichkeit:

Ezetimibe: (LDL‑C ca. 15 % gesenkt) und PCSK9-Inhibitoren in maximaler Dosis (LDL-C > 50 % reduziert; Alirocumab 150 mg oder Evolocumab 140 mg jede zweite Woche s.c.). Zusätzlich stehen als neue Therapieoptionen Inclisiran 284 mg (alle 6 Monate, LDL‑C ca 50 % reduziert) und Bempedoinsäure 180 mg (LDL‑C ca 20 % reduziert) zur Verfügung, wobei für Inclisiran noch keine kardiovaskulären Endpunktdaten vorliegen.

## Adjuvante Therapie mit Icosapentaensäureethylester

Icosapentaensäureethylester ist ein stabiler Ethylester der Eicosapentaensäure (EPA), die zur Klasse der Omega-3-Fettsäuren zählt. In der untersuchten Dosierung konnte – als add-on zu einer vorbestehenden Therapie mit Statinen konnte bei Patienten mit Triglyceriden zwischen 135 und 499 mg/dl mit und ohne Diabetes eine signifikante Reduktion kardiovaskulärer Endpunkte beobachtet werden.

## Monitoring und Sicherheitslabor

Der Effekt einer eingeleiteten Therapie sollte nach 4–6 Wochen reevaluiert werden und als Basis einer etwaigen Therapieanpassung dienen. Bei stabiler Therapie sind Kontrollen alle 12 Monate anzustreben.

Laborchemische Nebenwirkungen (Muskel und Leber) sind bei Statin-Therapie sehr selten. CK, GOT, GPT, sollten vor Beginn einer Statintherapie gemessen werden. Eine Routinekontrolle der CK im Follow-up wird bei asymptomatischen Patienten nicht empfohlen.

Auf die Möglichkeit einer (extrem seltenen) symptomatischen Myopathie muss der Patient hingewiesen werden.

## Evidenzlage

Basis der Therapieempfehlungen sind die Leitlinien der ESC [1, 2], des AACE/ACE [3] sowie mehrere Metaanalysen der verfügbaren Statinstudien [4–6], wobei eine davon sich spezifisch auf Patienten mit Diabetes mellitus bezieht [5].

Diese Metaanalysen belegen auch den klaren Zusammenhang zwischen dem Ausmaß der LDL Senkung und der Reduktion des vaskulären Risikos.

Die Evidenz zur Kombination von Ezetimibe mit einem Statin stammt aus der IMPROVE-IT Studie [7]. In dieser konnte bei Patienten nach einem akuten Koronarsyndrom durch die Kombination im Vergleich zu einer Monotherapie mit Statinen eine signifikante weitere Reduktion kardiovaskulärer Ereignisse beobachtet werden.

Die Evidenz zu PCSK9-Hemmern auf Basis einer Statintherapie stammt aus der FOURIER Studie (Evolocumab) [8] und deren Subanalysen, in denen sich Patienten mit Diabetes mellitus nicht von denen ohne Diabetes in ihren klinischen Vorteilen durch die Behandlung unterschieden. Ergänzend dazu gibt es Evidenz aus der ODYSSEY OUTCOMES Studie (Alirocumab) [9], in der ebenfalls 29 % der Population zu Studienbeginn an Diabetes erkrankt waren.

Die Evidenz zur Therapie mit Fibraten stammt aus der VAHIT Studie, in der eine Subgruppe von 627 Menschen mit Diabetes untersucht wurde [10], sowie aus (post-hoc) Analysen der FIELDS Studie [11] und der ACCORD Studie [12]. In den beiden letzteren wurde Fenofibrat meist „on top“ der Statintherapie eingesetzt. Für Bempedoinsäure liegt nun eine positive Outcomes Studie mit Reduktion des primären Endpunktes (MACE) vor (Clear Outcomes).

Die Evidenz zu Icosapentaensäureethylester stammt aus der REDUCE-IT Studie an mit Statinen vorbehandelten Patienten, mit und ohne Diabetes, welche einen Triglyceridspiegel von 135–499 mg/dl aufwiesen [13].

Für die Kombination verschiedener Lipidsenker gibt es mit Ausnahme der oben angeführten Studien zurzeit ausschließlich die pathophysiologischen Grundlagen und epidemiologischen Daten als Evidenz.

## Footnotes

1 Ohne zusätzliche Risikofaktoren oder Endorganerkrankung.

2 Mit zusätzlichen Risikofaktoren oder Endorganerkrankung.

## Interessenkonflikt

T.C. Wascher hat von folgenden Unternehmen Forschungsunterstützungen und/oder Honorare erhalten: Amgen, Astra Zeneca, MSD, Novartis, Sanofi, Daiichi-Sankyo. B. Paulweber hat von folgenden Unternehmen Forschungsunterstützungen und/oder Honorare erhalten: Amgen, Astra Zeneca, Bayer, Boehringer-Ingelheim, Lilly, MSD, Mylan, Novo Nordisk, Sanofi. H. Toplak hat von folgenden Unternehmen Forschungsunterstützungen und/oder Honorare erhalten: Amgen, Astra Zeneca, Mylan, MSD, Novonordisk, Pfizer, Sanofi. C.H. Saely hat von folgenden Unternehmen Forschungsunterstützungen und/oder Honorare erhalten: Amgen, Astra Zeneca, MSD, Mylan, Novartis, Sanofi. H. Drexel hat Honorare für Vorträge, Forschungsunterstützung und Beratungsaktivitäten von NovoNordisk, Merck, MSD, Sanofi, Bayer, Amgen, Boehringer Ingelheim, Servier erhalten. B. Föger hat von folgenden Unternehmen, die teilweise auch fördernde Mitglieder der ÖDG sind, Forschungsunterstützungen und/oder Honorare erhalten: Alexion, Amgen, AOP Orphan, Astra-Zeneca, Böhringer-Ingelheim, Janssen, Lilly, MSD, Novartis, Novo-Nordisk, Sanofi, Takeda. F. Hoppichler hat von folgenden Unternehmen Forschungsunterstützungen und/oder Honorare erhalten: Amgen, Sanofi, Novartis, Daiichi-Sankyo. T. Stulnig hat von folgenden Unternehmen Forschungs‑/Reisekostenunterstützung und/oder Honorare erhalten: Alexion, Amgen, AstraZeneca, Boehringer-Ingelheim, Meda, Mylan, Novo Nordisk, Sanofi. H. Stingl legt offen, dass er von folgenden Unternehmen, die teils auch fördernde Mitglieder der ÖDG sind, Forschungsunterstützungen, Honorare für Vorträge und/oder Beratertätigkeit, bzw. Fortbildungsunterstützung im Rahmen der (dienst)rechtlichen Rahmenbedingungen (Einladung zu Kongressen) erhalten hat: Amgen, AstraZeneka, Boehringer Ingelheim, Eli Lilly, Gebro, Menarini, MSD, Novo Nordisk, Novartis, Sanofi Aventis, Servier, Takeda, M. Clodi hat von folgenden Unternehmen Forschungsunterstützungen und/oder Honorare erhalten: Amgen, Astra Zeneca, MSD, Pfizer, Sanofi.
